# The role of mitochondria-related key genes in primary biliary cholangitis was analyzed based on transcriptome sequencing data

**DOI:** 10.3389/fimmu.2025.1644682

**Published:** 2025-10-09

**Authors:** Yanping Tao, Lei Zhong, Qihua Shen, Xiaofan Zhang, Xuyun Xu, Runlin Feng

**Affiliations:** ^1^ Department of Emergency Medicine, Kunming Third People’s Hospital, Kunming, Yunnan, China; ^2^ Department of Hepato-Pancreato- Surgery, Huzhou Central Hospital, Zhejiang, Huzhou, China; ^3^ Department of Pathology, The Second Affiliated Hospital of Kunming Medical University, Kunming, Yunnan, China

**Keywords:** primary biliary cholangitis, SHANK2, TGM2, mitochondria, bioinformatics

## Abstract

**Introduction:**

Mitochondrial dysfunction is implicated in the pathogenesis of primary biliary cholangitis (PBC), but the roles of mitochondria-related genes (MRGs) remain unclear. We aimed to identify key MRGs associated with PBC and validate their expression at transcriptional and protein levels.

**Methods:**

Peripheral blood from PBC patients and healthy controls underwent RNA-seq. MRGs were sourced from MitoCarta 3.0. After quality control, differentially expressed genes were defined between PBC and controls and integrated with weighted gene co -expression network analysis to obtain candidate genes. LASSO and SVM-RFE selected hub genes, whose diagnostic performance was assessed by ROC in both the discovery cohort and an external validation dataset. Functional enrichment, immune-cell composition analyses, and regulatory network construction (miRNA/lncRNA/TF) were performed. Protein and mRNA expression were validated by liver -tissue immunohistochemistry and peripheral-blood RT-qPCR, respectively. Disease correlation and drug-prediction analyses were conducted.

**Results:**

SHANK2 and TGM2 were identified as hub MRGs, showed higher expression in PBC, and achieved AUC values >0.7. Enrichment linked SHANK2 to Bcell receptor, T-cell receptor, and Wnt signaling pathways, while TGM2 associated with oxidative phosphorylation and nucleotide metabolism. Immune-cell profiles differed between PBC and controls, and selected cell types correlated with hub-gene expression. Regulatory analyses suggested modulation of SHANK2 and TGM2 by molecules including SLFN12L and DNAH10OS. Immunohistochemistry revealed elevated, stage-associated protein expression of both genes in PBC liver tissues, and RT-qPCR confirmed higher peripheralblood mRNA levels. Drug/disease analyses indicated therapeutic potential for targeting these genes.

**Discussion:**

SHANK2 and TGM2 emerge as key MRGs linking mitochondrial dysfunction with immune dysregulation in PBC and may serve as diagnostic biomarkers and therapeutic targets; further mechanistic and clinical validation is warranted.

## Introduction

Primary biliary cholangitis (PBC), a chronic autoimmune disorder affecting the liver, is marked by gradual intrahepatic bile duct destruction, leading to cholestasis, fibrotic progression, and eventual cirrhosis (1). Clinical manifestations encompass fatigue, pruritus, and jaundice, with severe cases potentially progressing to liver failure (2). Mitochondrial antigen-specific autoimmunity (particularly targeting PDC-E2), combined with genetic predisposition and environmental influences, constitutes the tripartite etiological basis of PBC (3). Current therapeutic options, including ursodeoxycholic acid (UDCA) and obeticholic acid, can delay disease progression but are ineffective in approximately 30-40% of patients, highlighting the critical need for novel therapeutic strategies (4). Identification of key molecular drivers, especially those associated with mitochondrial dysfunction, may provide valuable insights into the pathogenesis of PBC and inform the development of precision therapies.

Mitochondria are essential organelles that play a pivotal role in energy production, cellular metabolism, and the regulation of apoptosis. They are critical for maintaining cellular homeostasis and are involved in numerous cellular processes, such as oxidative phosphorylation, calcium homeostasis, and modulation of apoptosis (5). Dysregulation of mitochondrial function has been implicated in several diseases, including metabolic disorders, neurodegenerative conditions, and autoimmune diseases (6). In the context of PBC, mitochondrial dysfunction is hypothesized to contribute to the loss of tolerance to mitochondrial antigens, a central feature of the autoimmune component of the disease (7). Previous studies have demonstrated that mitochondrial damage and the presence of antimitochondrial antibodies are key factors in the pathogenesis of PBC; however, the precise molecular mechanisms underlying these associations remain largely elusive. Although some studies have investigated mitochondria-related genes (MRGs) in PBC, the full extent of their involvement and the identification of key genes remain underexplored. Therefore, the investigation of key MRGs in PBC is crucial to elucidate the underlying mechanisms of the disease and to identify potential therapeutic targets.

This study integrates transcriptomic profiling and bioinformatics to identify mitochondrial-associated hub genes in PBC. Utilizing blood-derived RNA sequencing data from 15 PBC patients and 15 healthy controls, we delineated differentially expressed mitochondrial-related genes (MRGs), validated their diagnostic utility through machine learning techniques, and explored their functional networks, immune correlations, and regulatory mechanisms. Our analysis identified SHANK2 and TGM2 as key MRGs enriched in immune-metabolic pathways, demonstrating strong diagnostic accuracy with an AUC greater than 0.7. Furthermore, we mapped their interactions with non-coding RNAs, transcription factors, and immune cells, while predicting repurposable drugs targeting these genes. These findings provide a comprehensive roadmap for the development of MRG-based biomarkers and therapeutic strategies, effectively bridging the gap between mitochondrial biology and the pathogenesis of PBC.

## Materials and method

### Data collection

All liver tissue and peripheral blood samples used in this study were collected from the Second Affiliated Hospital of Kunming Medical University, with a collection period from January 2020 to December 2024. The samples included 40 liver biopsy samples from patients with PBC and 40 liver biopsy samples from non-PBC patients. In the PBC patient group, subgroup analysis was further performed according to pathological staging. Additionally, peripheral blood samples were obtained from 15 PBC patients and 15 healthy controls. All samples were approved by the Ethics Committee of the Second Affiliated Hospital of Kunming Medical University (Approval Number: Review-PJ-Department-2023-296), and informed consent was obtained from all patients. All samples were stored and managed in compliance with the hospital’s biobank regulations. The inclusion criteria were as follows: age ≥ 18 years; meeting the EASL 2017 diagnostic criteria for primary biliary cholangitis, including at least two of the following indicators: elevated ALP (≥1.5 times the upper limit of normal), positive AMA or AMA-M2 antibodies, and liver tissue pathology showing small bile duct damage and portal inflammatory infiltration. The exclusion criteria were as follows: coexisting viral hepatitis, autoimmune hepatitis, tumors, other severe systemic diseases, and pregnancy, as well as other pathological conditions unrelated to the study. All samples were obtained from patients at the Second Affiliated Hospital of Kunming Medical University, ensuring the representativeness and consistency of the experimental samples.

**Supplementary Table 1** provides details of the 15 PBC patients and 15 normal blood samples used for transcriptome sequencing to create the training set. The GSE119600 dataset (based on the GPL10558 platform) was also downloaded from the Gene Expression Omnibus (GEO; https://www.ncbi.nlm.nih.gov/geo/). This dataset comprises whole blood samples from 90 PBC patients and 47 controls and was used as the validation set for this study. A total of 1,949 MRGs were downloaded from MitoCarta 3.0 database (8).

### Transcriptome sequencing and data analysis

Data used for sequencing were evaluated by FastQC package (version 0.11.9) (9). Before analysis, raw sequencing data underwent quality control preprocessing to eliminate adapter sequences and low-quality reads. The resulting clean reads were subsequently aligned to the murine reference genome using HISAT2 (v2.2.1) (10) with standard parameters. Transcript and gene expression quantification were performed using StringTie’s FPKM normalization method. This approach accounted for both mapped read counts and transcript length variations across samples. Expression pattern disparities were subsequently visualized through principal component analysis (PCA). The DESeq2 package (version 1.36.0) (11) was used to identify DEGs between PBC and control samples. Screening criteria were p < 0.05 and |log_2_FoldChange (FC)| > 1. Then ggplot2 (version 3.3.5) (12) and pheatmap package (version 1.0.12) (13) were used to draft volcano map and heatmap to display DEGs.

### Weighted gene co-expression network analysis

For further accessing genes associated with PBC, WGCNA was conducted using the WGCNA package (version 1.70-3) (14). Initially, hierarchical clustering of all samples was executed to investigate outliers and determine whether their exclusion was necessary to ensure subsequent analyses’ accuracy. Network construction parameters were optimized by selecting a β value that satisfied dual criteria (1): scale-free topology fit (R² ≥ 0.85) and (2) minimal average connectivity. Gene relationship analysis then proceeded through pairwise similarity computation, culminating in phylogenetic tree construction via average linkage hierarchical clustering. Module detection was performed through dynamic tree cutting algorithm implementation, enforcing a minimum module size of 100 genes. Modules demonstrating the strongest association (highest correlation coefficients) with phenotypic traits were selected as pivotal modules, whose member genes were classified as biologically relevant candidates.

### Identification and analysis of candidate genes

By taking the intersection of DEGs, key module genes and MRGs, candidate genes were obtained. To find potential functions and pathways enriched by candidate genes, GO and KEGG enrichment analyses were performed by clusterProfiler package (p < 0.05 and count ≥ 1) (version 4.0.2) (15).

### Screening of hub genes

LASSO and SVM-RFE were carried out to screen signature genes. Interaction of signature genes derived from 2 machine-learning algorithms was identified as candidate hub genes. Receiver operating characteristic (ROC) curves were then plotted for all samples in the training and validation cohorts using the pROC package (version 4.0.2) to evaluate the ability of the candidate hub genes to distinguish between the PBC patient group and the control group (16). Genes with an area under the curve (AUC) of over 0.7 in both the training and validation sets were selected as the final hub genes and their expression levels were analyzed. Furthermore, correlation between hub genes was explored by spearman correlation analysis. Cell-PLoc 2.0 performed subcellular localization on proteins of hub genes (17).

### Enrichment and function analysis

In order to explore the biological function of hub genes, GSEA was carried out. Background gene set c5.go.v7.4.entrez.gmt and c2.cp.kegg.v7.4.entrez.gmt were downloaded from GSEA website. Samples were divided into high and low expression groups based on expression median value of hub genes. GSEA of all genes in 2 expression groups was processed with threshold |NES| > 1, NOM P < 0.05 and q < 0.25. Back-propagation neural network (BPNN) was constructed by neuralnet package (version 4.0.2) (18) to evaluate accuracy of hub genes.

The similarity of GO terms of hub genes was calculated by GOSemSim package (version 2.18.1) (19).

### Immune infiltration analysis

To analyze differences in the immune microenvironment between the PBC and control groups, CIBERSORT (version 0.1.0) was used to evaluate the infiltration levels of 22 immune cell subpopulations (20) Gene and immune cell expression matrices were processed using a deconvolution algorithm to obtain an immune infiltration profile, visualized using ggplot2 (version 3.3.5). To identify immune cells showing significant differences between the two groups, we analyzed differences in immune cell composition between samples using the Wilcoxon test, setting p < 0.05 as the significance threshold. The results were visualized using ggplot2 (version 3.3.5).

### Construction of regulatory network

MicroRNAs (miRNAs)-targeting hub genes predicted through miRDB (http://mirdb.org) and miRWalk database. Key miRNAs were acquired by overlapping miRNAs obtained from the 2 databases (energy < -30). Then, lncRNAs-targeting miRNAs were obtained from the miRTarBase database. Finally, Cytoscape software was used to visualize the lncRNAs-miRNA-mRNA network. To further understand the regulatory effects of hub genes in disease, TFs-targeting hub genes were predicted using Cistrome database (RF score > 0.75), and the TF-mRNA-miRNA network was constructed to visualize potential relationships.

Correlated diseases of hub genes were analyzed by DisGeNET database (http://www.disgenet.org/), and the hub genes-diseases co-expression network was visualized by NetworkAnalyst (http://www.networkanalyst.ca). Based on hub genes, small molecule drugs were found by DGIdb.

### Immunohistochemical analysis

From January 2018 to October 2024, a total of 30 paraffin-embedded liver tissue samples were collected from patients diagnosed with primary biliary cholangitis (PBC) at the Department of Pathology, The Second Affiliated Hospital of Kunming Medical University. All diagnoses were confirmed by pathological examination. For comparison, 17 normal liver tissue samples were used as controls. PBC patients were stratified into four histological stages according to Ludwig’s classification. Immunohistochemical staining was performed to assess the protein expression of SHANK2 and TGM2. The staining protocol strictly followed the instructions provided by the antibody manufacturers. Sections were deparaffinized, rehydrated, and subjected to antigen retrieval, followed by incubation with primary antibodies against SHANK2 and TGM2, respectively. Signal detection was achieved using an HRP-conjugated secondary antibody and DAB chromogen. Expression levels were semi-quantitatively assessed based on staining intensity and percentage of positive cells. Comparisons between groups and different stages were conducted using appropriate statistical methods, with p-values < 0.05 considered significant.

### Quantitative real-time PCR

Peripheral blood samples were obtained from 10 patients with PBC and 8 healthy controls. Peripheral blood mononuclear cells (PBMCs) were isolated using Ficoll density gradient centrifugation. Total RNA was extracted from PBMCs using TRIzol reagent, and the RNA concentration and purity were determined using a Nanodrop spectrophotometer. Complementary DNA (cDNA) was synthesized using a reverse transcription kit, and qPCR was performed using SYBR Green Master Mix on a real-time PCR system. The relative mRNA expression levels of SHANK2 and TGM2 were quantified using the 2^−ΔΔCt method and normalized to GAPDH as an internal control. Statistical comparisons between groups were conducted using a t-test, and a p-value of < 0.05 was considered statistically significant.

### Statistical analysis

All statistical analyses were conducted in R software and Microsoft Excel. The Wilcoxon test and t test were utilized to assess the differences between different groups. The P value less than 0.05 was considered statistically significant.

## Results

### Totally 1,905 DEGs were identified between PBC and control groups

**Figure 1:**
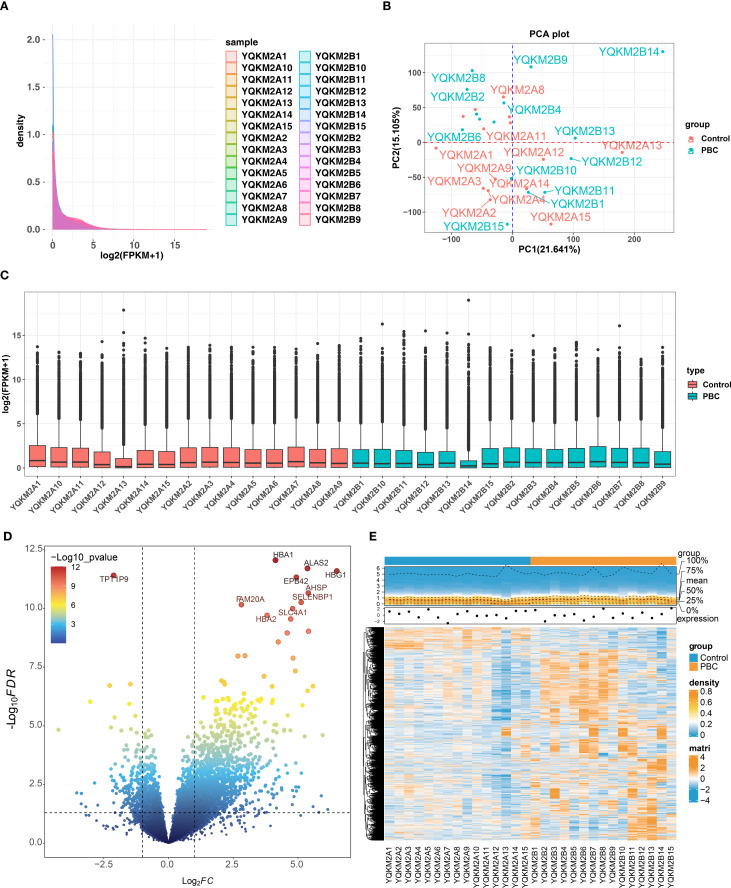
Quality control and differential-expression overview for RNA-seq in PBC vs. controls. **(A)** Density distribution of gene expression (log2(FPKM+1) across all 30 samples (15 PBC, 15 controls), showing consistent overall patterns. **(B)** PCA plot indicating separation between PBC and control groups. **(C)** Boxplots of normalized expression after QC, demonstrating comparable distributions across samples. **(D)** Volcano plot of DEGs identified by DESeq2 (p < 0.05 and |log2FC| > 1); in total 1,905 DEGs, including 1,706 upregulated and 199 downregulated genes. **(E)** Heatmap of DEGs revealing distinct expression signatures between groups. Note: Read alignment rate exceeded 90% and base error rate was <1/1000 (see **Supplementary Table**).

Alignment rate of 30 sequencing samples was all above 90% through alignment analysis and base error rate was less than 1/1000 (**Supplementary Table 2**). And FPKM distribution of each samples showed that sample sequencing effect was decent (**Figure 1A**). PCA diagram and Boxplot showed that overall sequencing quality was high (**Figures 1B-C**). Furthermore, 1,905 DEGs were identified between PBC and control samples, of which 1,706 up-regulated and 199 down-regulated (**Figures 1D-E**).

### Totally 7 candidate genes were identified and analyzed

**Figure 2:**
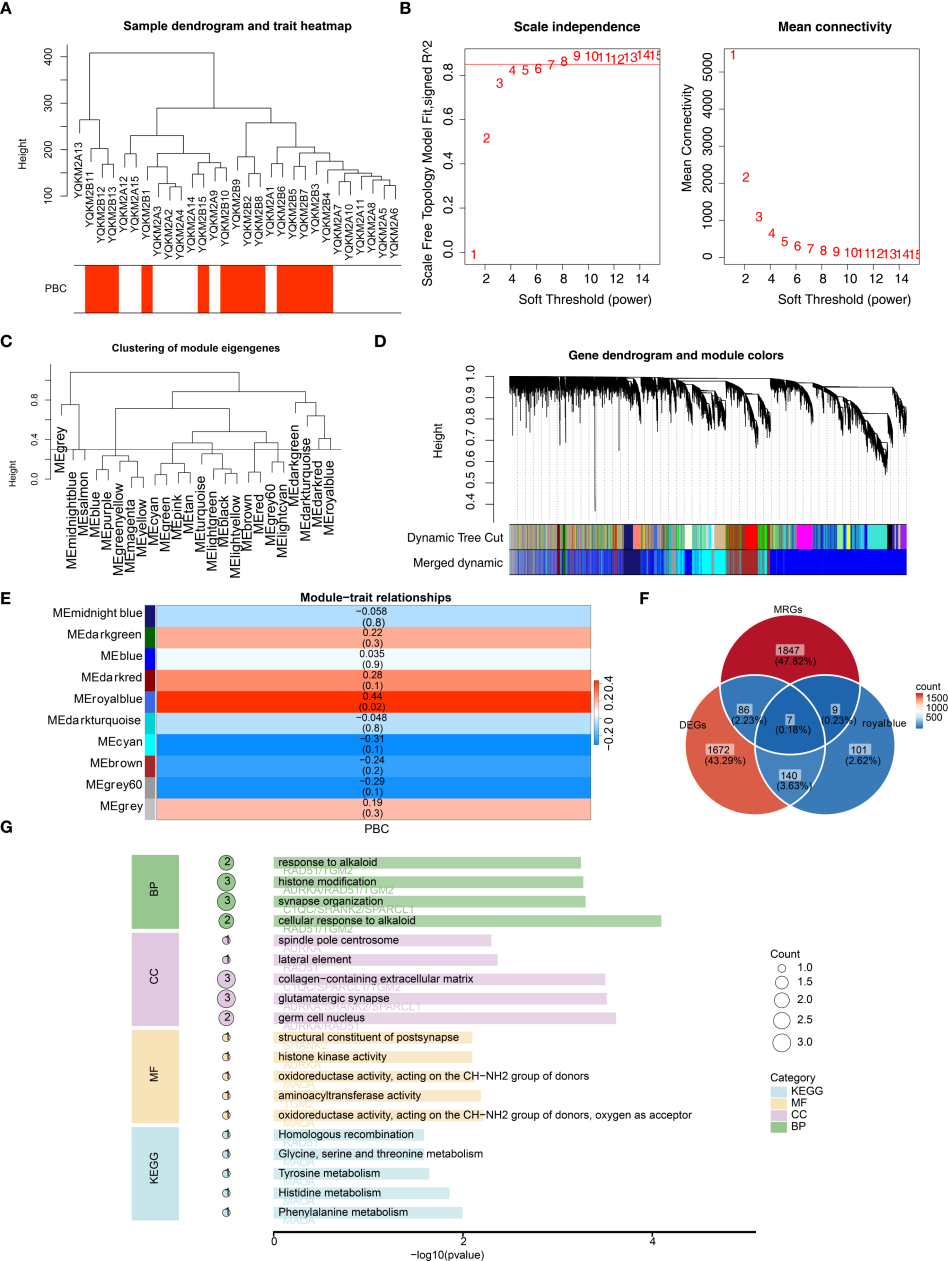
WGCNA identifies a PBC-associated module and mitochondria-related candidate genes, with functional enrichment. **(A)** Sample clustering dendrogram with trait heatmap (PBC status). One outlier sample (YQKM2B14) was identified and removed from downstream analyses. **(B)** Soft-threshold power selection for scale-free topology: β = 9 was chosen (scale-free topology fit R^2^ > 0.85; mean connectivity ~0). **(C)** Clustering of module eigengenes showing relationships among co-expression modules. **(D)** Gene dendrogram with dynamic tree cut and merged modules; a total of 10 co-expression modules were obtained (minimum module size = 100 genes). **(E)** Module–trait relationships heatmap. The MEroyalblue module shows the strongest positive correlation with PBC (|r| = 0.44, P < 0.05). **(F)** Venn diagram intersecting DEGs (n = 1,905), MEroyalblue genes (n = 257), and mitochondria-related genes (MRGs, n = 1,949), yielding 7 candidate genes: AURKA, C1QC, MAOA, RAD51, SHANK2, SPARCL1, and TGM2. **(G)** GO and KEGG enrichment of the 7 candidate genes. Top enriched terms indicate associations with histone modification, transaminase/oxidoreductase activity, and tyrosine metabolism (representative top terms across BP/CC/MF and KEGG are displayed; bar length denotes –log10(P-value), point size denotes gene count).

We performed WGCNA to identify key module genes associated with PBC. Based on hierarchical clustering of all sequencing samples, one outlier (YQKM2B14) was detected and excluded from downstream analyses (**Figure 2A**). Subsequently, we selected the optimal soft-thresholding power (β = 9), which yielded a scale-free topology fit index R² greater than 0.85 with mean connectivity close to zero, indicating that the constructed network closely approximated a scale free topology (**Figure 2B**). Using this parameter, a gene co-expression network was established and a total of 10 distinct modules were identified, each containing at least 100 genes (**Figure 2C**). Lastly, Module–trait relationship analysis revealed that the MEroyalblue module showed the strongest positive correlation with PBC(|cor| = 0.44, P < 0.05), and 257 genes within this module were considered key module genes (**Figures 2D, E**). Byoverlapping the 1,905 DEGs, the 257 MEroyalblue module genes, and 1,949 mitochondria-related genes (MRGs), weidentified seven candidate genes: AURKA, C1QC, MAOA, RAD51, SHANK2, SPARCL1, and TGM2 (**Figure 2F**; **Supplementary Table 3**). To further explore the biological roles of these candidate genes, GO and KEGG enrichment analyses were performed. In total, 4 biological process (BP), 12 cellular component (CC), 17 molecular function (MF), and 13 KEGG pathways were significantly enriched. The top representative terms included histone modification, synapse organization, oxidoreductase activity, and tyrosine metabolism **Figure 2G**, These findings indicate that the candidate genes are functionally linked to epigenetic regulation, energy metabolism, and amino acid metabolism, suggesting their potential involvement in the pathogenesis of PBC.

### SHANK2 and TGM2 were final hub genes

**Figure 3:**
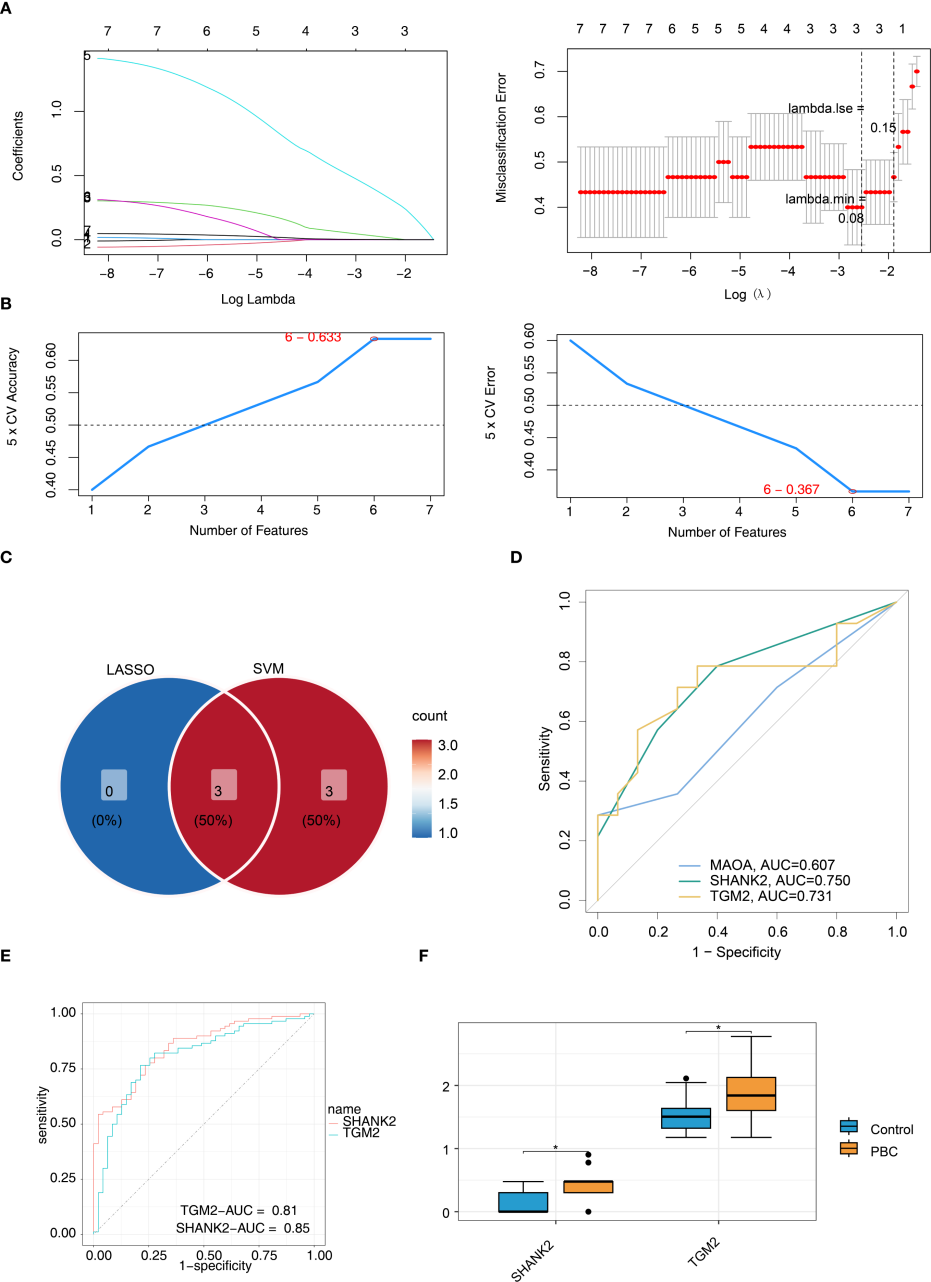
Identification of SHANK2 and TGM2 as final hub genes using machine learning and validation. **(A)** LASSO regression identified three candidate genes (MAOA, SHANK2, and TGM2) at lambda.min = 0.08. **(B)** SVM-RFE algorithm selected six candidate genes (TGM2, AURKA, C1QC, MAOA, SHANK2, and RAD51) with maximal accuracy (0.633) and minimal RMSE (0.367). **(C)** Intersection of LASSO and SVM-RFE results yielded three overlapping genes (MAOA, SHANK2, and TGM2), which were considered candidate hub genes. **(D)** ROC curves for candidate genes in the training set showed SHANK2 and TGM2 achieved AUC values above 0.7, while MAOA had lower diagnostic performance (AUC = 0.607). **(E)** ROC validation further confirmed the diagnostic value of SHANK2 (AUC = 0.85) and TGM2 (AUC = 0.81). **(F)** Boxplots revealed significantly higher expression levels of SHANK2 and TGM2 in PBC patients compared with controls.

LASSO regression algorithm was used to select MAOA, SHANK2 and TGM2 as signature genes (lambda.min = 0.08) (**Figure 3A**). Then SVM-RFE algorithm screened 6 signature genes2 (TGM2, AURKA, C1QC, MAOA, SHANK2 and RAD51) when maximal accuracy was 0.633, minimal RMSE was 0.367 (**Figure 3B**). Intersection of signature genes (MAOA, SHANK2 and TGM2) was taken and identified as candidate hub genes (**Figure 3C**). To evaluate these hub genes’ ability to distinguish PBC from control samples, ROC curves were plotted for all samples in the training and validation sets. The results showed that the AUC for both SHANK2 and TGM2 exceeded 0.7, whereas the AUC value for MAOA was 0.607 in the training set. Thus, SHANK2 and TGM2 were obtained as hub genes for subsequent analysis (**Figures 3D, E**). Expression of SHANK2 and TGM2 was higher in PBC group (**Figure 3F**).

### Function analysis of SHANK2 and TGM2

**Figure 4:**
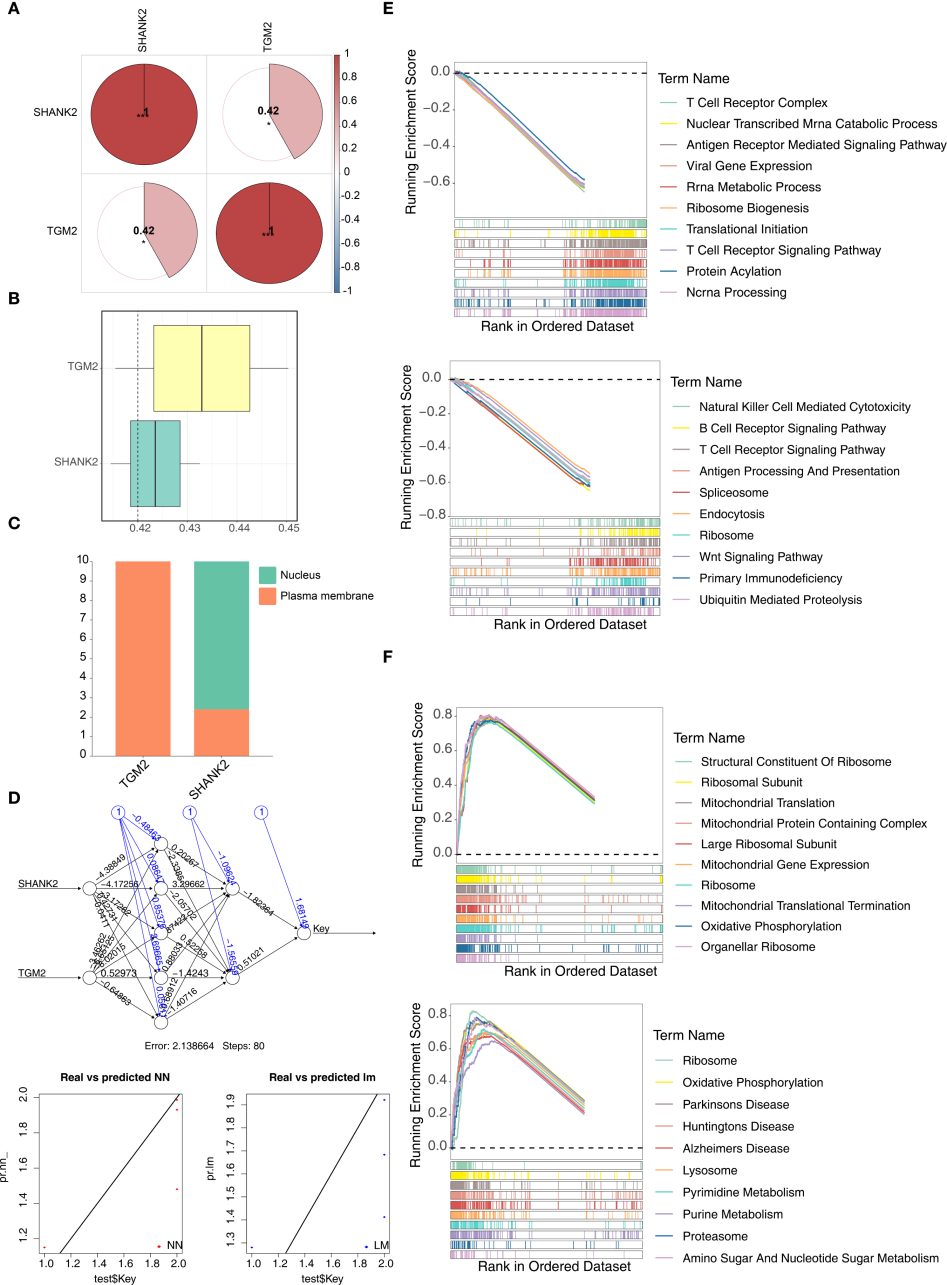
Functional analyses of hub genes SHANK2 and TGM2. **(A)** Spearman correlation analysis revealed a significant positive correlation between SHANK2 and TGM2 (r = 0.42, p < 0.05). **(B)** Similarity analysis showed that SHANK2 and TGM2 had a similarity score >0.4, indicating strong similarity. **(C)** Subcellular localization predicted that TGM2 is mainly distributed on the plasma membrane, while SHANK2 is located in both the nucleus and plasma membrane. **(D)** A back propagation neural network (BPNN) was constructed based on the hub genes; the prediction correlation was 0.8199 with a mean error of 0.1326, suggesting good predictive performance. **(E)** GSEA results indicated that SHANK2 was enriched in 921 GO terms and 56 KEGG pathways, mainly including B cell receptor signaling, T cell receptor signaling, and Wnt signaling pathways. **(F)** GSEA showed that TGM2 was enriched in 1364 GO terms and 74 KEGG pathways, including oxidative phosphorylation, pyrimidine metabolism, and purine metabolism.

Spearman correlation analysis was used to determine whether there was correlation between hub genes. Results indicated that SHANK2 and TGM2 had a significant positive correlation (r = 0.42, p < 0.05) (**Figure 4A**). Similarity analysis showed that the similarity score of SHANK2 and TGM2 was greater than 0.4, which indicated that hub genes had strong similarity (**Figure 4B**). Subcellular localization showed TGM2 was mainly expressed in the plasma membrane, while SHANK2 was mainly expressed in nucleus and plasma membrane (**Figure 4C**). Based on hub genes, a BPNN was constructed and prediction correlation and error respectively were 0.8199 and 0.1326, indicating a small prediction bias of the BPNN model (**Figure 4D**). Through GSEA, SHANK2 correlated with 921 GO terms and 56 KEGG pathways which mainly were B cell receptor signaling pathway, T cell receptor signaling pathway and wnt signaling pathway (**Figure 4E**). TGM2 enriched in 1364 GO terms and 74 KEGG pathways, including oxidative phosphorylation, pyrimidine metabolism and purine metabolism (**Figure 4F**).

### Immune microenvironment played a crucial role in PBC patients

**Figure 5:**
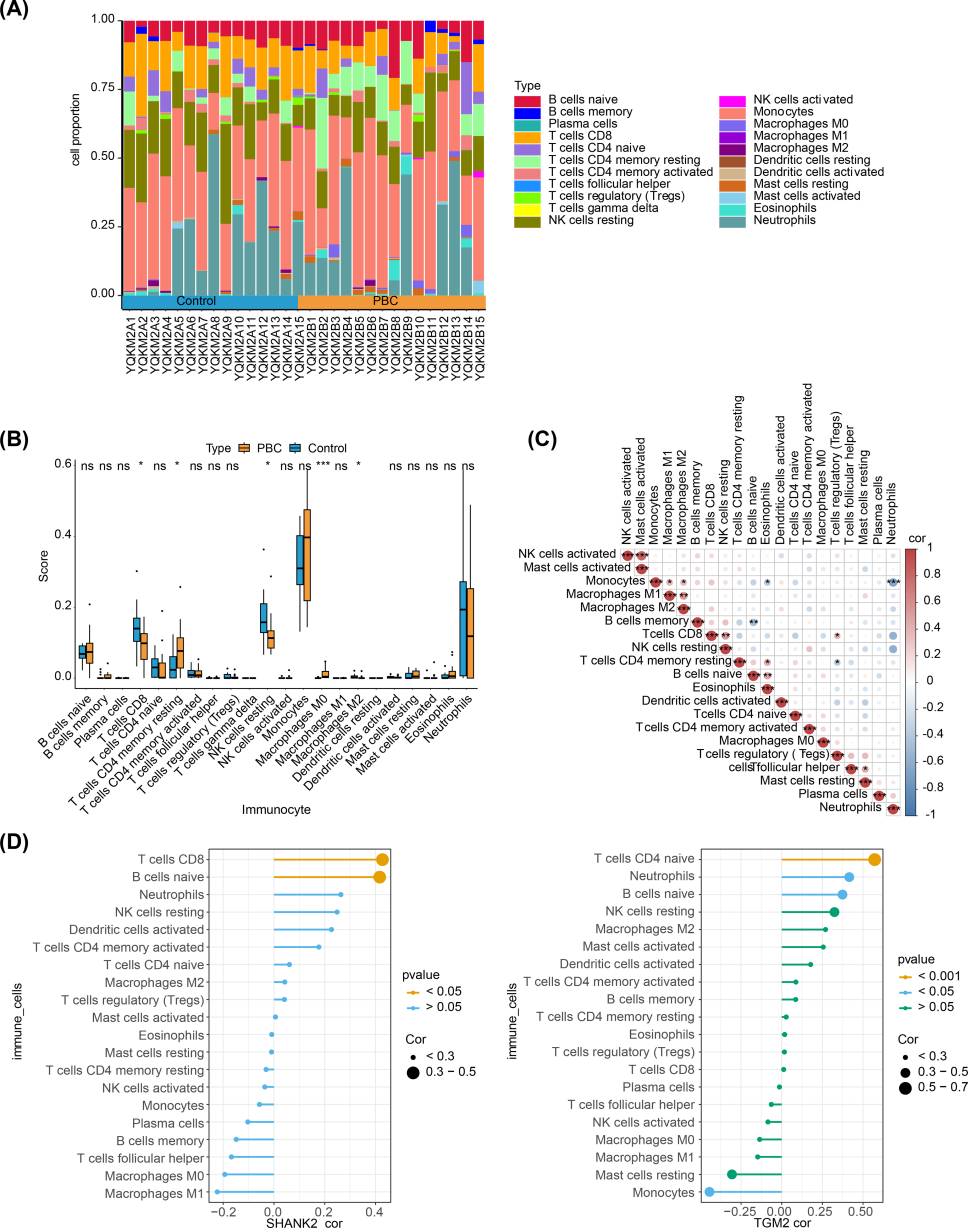
Immune microenvironment analysis in PBC and its association with hub genes. **(A)** Stacked bar plots showing the proportion of immune infiltrating cells in PBC and control samples. **(B)** Boxplots of immune cell abundance revealed significant differences between groups: CD8+ T cells, resting NK cells, and M2 macrophages were enriched in controls, whereas central memory CD4+ T cells and undifferentiated M0 macrophages were enriched in PBC. **(C)** Correlation heatmap of immune cells. Activated NK cells were positively correlated with activated mast cells (r > 0.4, p < 0.05), while monocytes were negatively correlated with neutrophils (r < –0.4, p < 0.05). **(D)** Correlation analysis of hub genes with differential immune cells. SHANK2 was positively correlated with M0 and M1 macrophages, whereas TGM2 showed a significant positive correlation with monocytes.

The percentage abundance of immune infiltration cells for PBC and control samples is shown in **Figure 5A**. Immune profiling identified five differentially abundant leukocyte subsets between cohorts. The control group demonstrated CD8+, dormant NK cells, and M2 macrophages, contrasting with PBC specimens, which displayed enrichment of central memory CD4+ T cells and undifferentiated M0 macrophages (**Figure 5B**). In these immune cells, activated NK cells positively correlated with activated mast cells (r > 0.4, p < 0.05), while monocytes were negatively related with neutrophils (r < -0.4, p < 0.05) (**Figure 5C**). Finally, hub genes correlated with differential immune cells. SHANK2 was positively related to M0 and M1 macrophages, while TGM2 had significant positive correlation with monocytes (**Figure 5D**).

### Regulatory network analysis

**Figure 6:**
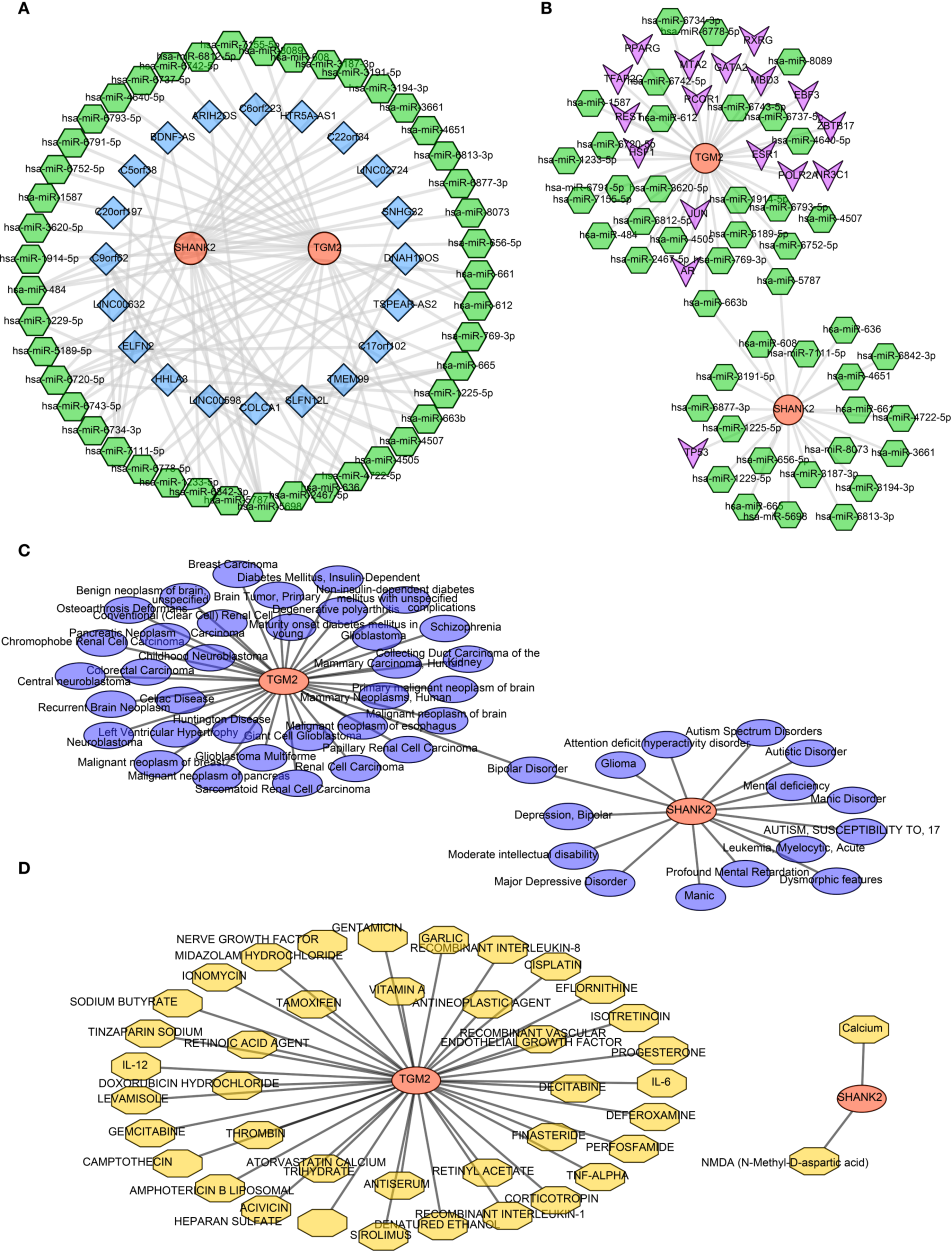
Regulatory network analyses of hub genes SHANK2 and TGM2 **(A)** Construction of the lncRNA–miRNA–mRNA network, including 45 key miRNAs and 21 lncRNAs. SHANK2 was regulated by SLFN12L, COLCA1, and LINC00598 through hsa-miR-665, while TGM2 was regulated by DNAH10OS, SLFN12L, TSPEAR-AS2, and HTR5A-AS1 via hsa-miR-6720-5p. **(B)** TF–mRNA–miRNA regulatory network. Seventeen transcription factors were predicted; TFAP2C, REST, and PPARG regulated TGM2, whereas SHANK2 was regulated only by TP53. **(C)** Disease predictions by DisGeNET. A total of 49 diseases were associated with the hub genes, with bipolar disorder being predicted by both SHANK2 and TGM2. **(D)** Drug predictions based on hub genes. Forty-one small molecule drugs were identified, including 39 targeting TGM2 (e.g., cisplatin, vitamin A) and 2 targeting SHANK2 (calcium and NMDA).

In order to explore involved regulatory mechanism of hub genes, lncRNA-miRNA-mRNA network was constructed with 45 key miRNAs and 21 lncRNAs. SLFN12L, COLCA1 and LINC00598 regulated SHANK2 through hsa-miR-665, and DNAH10OS, SLFN12L, TSPEAR-AS2 and HTR5A-AS1 regulated TGM2 by hsa-miR-6720-5p (**Figure 6A**). Then 17 TFs were predicted, and TF-mRNA-miRNA network was constructed. In TF-mRNA-miRNA network, TFAP2C, REST, PPARG, etc regulated TGM2, while only TP53 regulated SHANK2 **(Figure 6B**). Based on hub genes, 49 diseases were predicted by DisGeNET, bipolar disorder was disease predicting by SHANK2 and TGM2 simultaneously (**Figure 6C**). In addition, 41 small molecule drug types were predicted by hub genes, 39 types-targeting TGM2 (CISPLATIN, VITAMIN A, etc) and 2 types-targeting SHANK2 (calcium, N-Methyl-D-aspartic acid (NMDA)) were included (**Figure 6D**).

### SHANK2 and TGM2 protein expression in liver tissues

**Figure 7:**
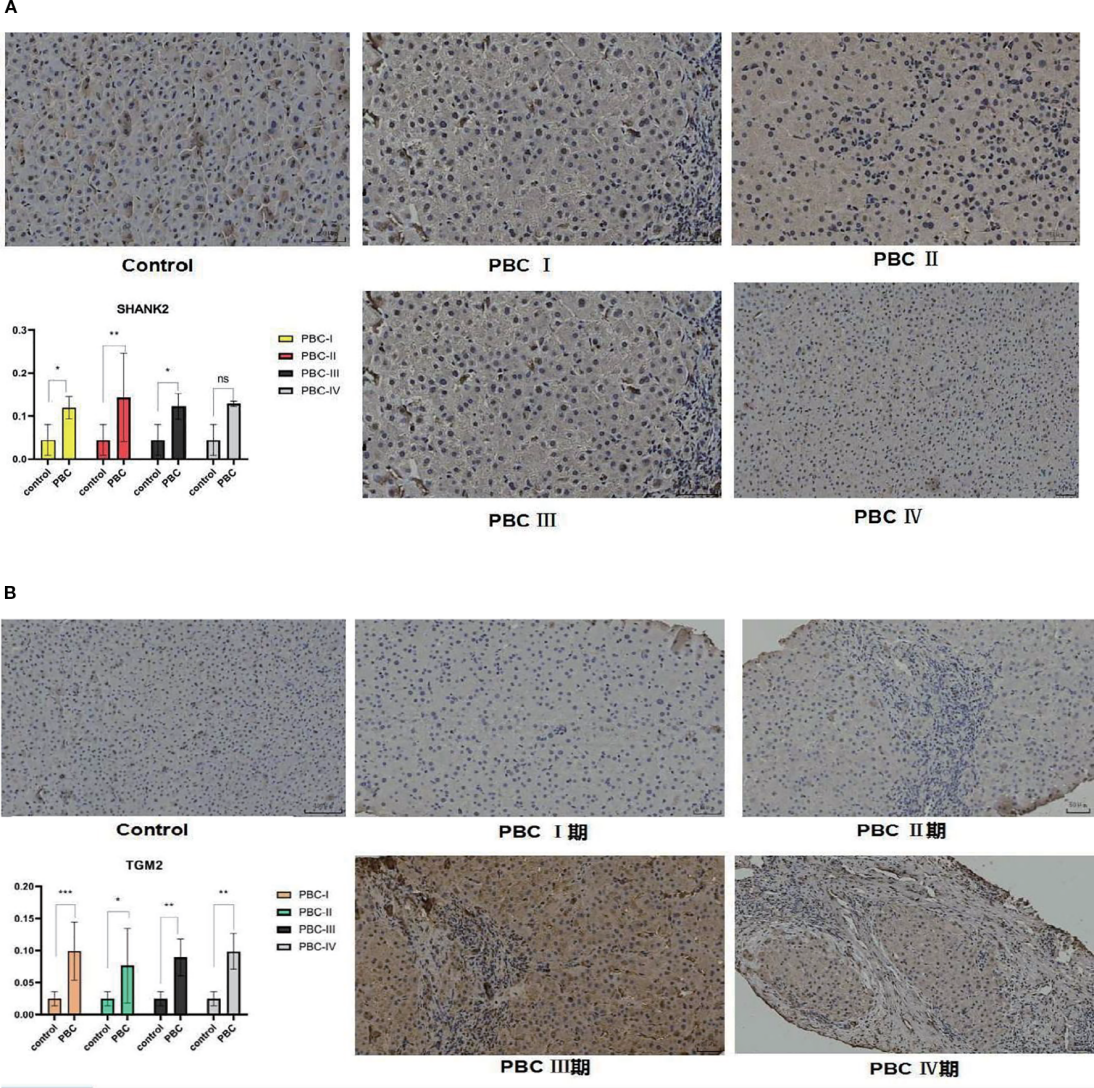
Immunohistochemical validation of SHANK2 and TGM2 protein expression in liver tissues of PBC patients. **(A)** Representative immunohistochemistry (IHC) images and quantitative analysis of SHANK2 expression in control and PBC liver tissues at different pathological stages (I–IV). SHANK2 was significantly upregulated in PBC stages I, II, and III compared with controls (P < 0.05). **(B)** Representative IHC images and quantitative analysis of TGM2 expression in control and PBC liver tissues. TGM2 expression was significantly elevated across all four stages of PBC compared with controls (P < 0.05). *P < 0.05; **P < 0.01; ***P < 0.001. 期, denotes the different stages of primary biliary cholangitis (PBC) (stage I, II, III, and IV), which are used to distinguish the pathological progression of the disease.

To further validate the bioinformatics findings at the protein level, immunohistochemistry was employed to evaluate SHANK2 and TGM2 expression in liver tissues. SHANK2 was significantly overexpressed in the liver tissues of PBC patients compared to the control group, with prominent expression differences observed in stages I, II, and III of PBC (P < 0.05) (**Figure 7A**). Similarly, TGM2 protein expression was markedly elevated in the PBC group, with statistically significant increases noted across all four pathological stages (P < 0.05) (**Figure 7B**). These results suggest that both SHANK2 and TGM2 proteins are upregulated in the progression of PBC and may be involved in stage-specific pathological processes.

### SHANK2 and TGM2 mRNA expression in peripheral blood

**Figure 8:**
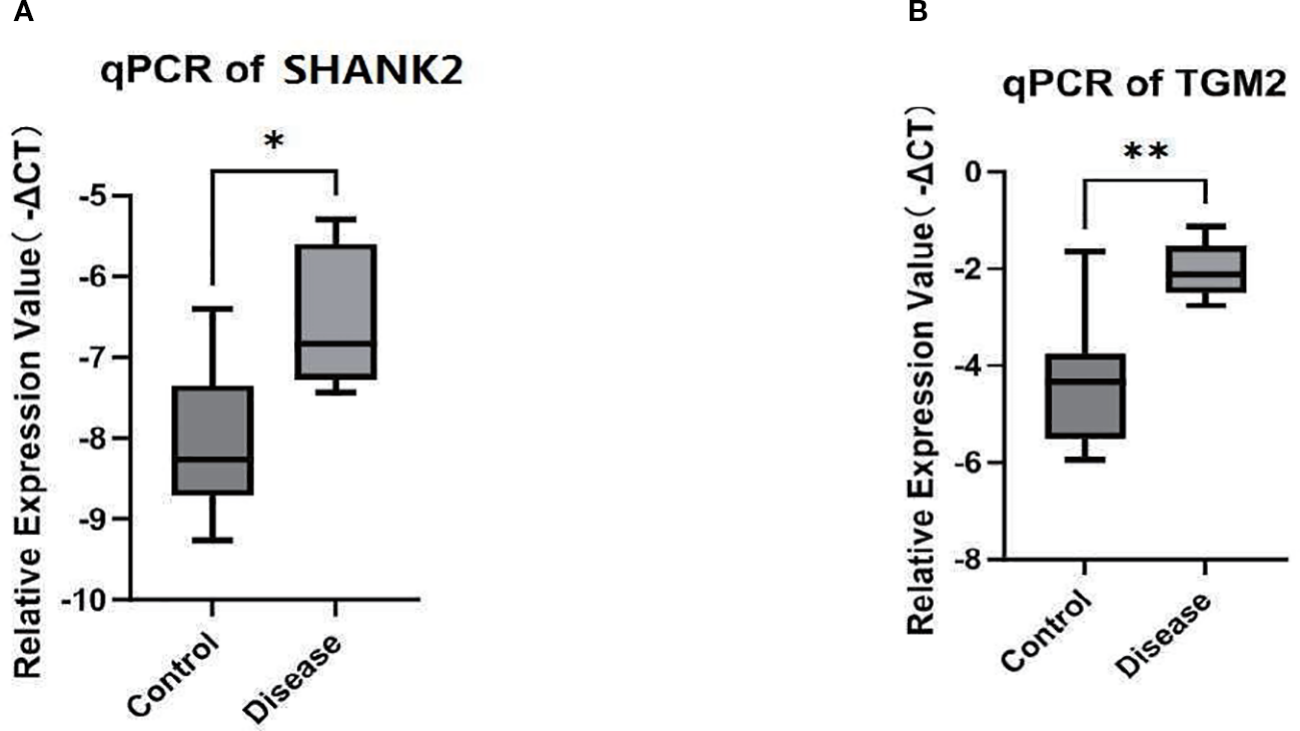
Validation of SHANK2 and TGM2 mRNA expression in peripheral blood. **(A)** Quantitative PCR (qPCR) analysis of SHANK2 expression in peripheral blood mononuclear cells (PBMCs) from PBC patients and healthy controls. SHANK2 expression was significantly elevated in the PBC group (P < 0.05). **(B)** qPCR analysis of TGM2 expression in PBMCs. TGM2 expression was markedly increased in the PBC group compared with controls (*P < 0.01; **P < 0.01).

To investigate SHANK2 and TGM2 expression at the transcriptional level, qPCR analysis was performed on RNA extracted from PBMCs of PBC patients and healthy controls. The results demonstrated significantly elevated mRNA expression levels of both SHANK2 and TGM2 in the PBC group compared to controls (P < 0.05) (**Figure 8A, B**). These findings confirm the upregulation of SHANK2 and TGM2 in PBC not only in liver tissue but also in peripheral immune cells, indicating a potential systemic involvement in disease pathogenesis.

## Discussion

PBC is an organ-specific autoimmune disorder targeting small-to-medium intrahepatic bile ducts, pathologically characterized by progressive nonsuppurative destructive cholangitis that inevitably progresses to ductopenia and secondary biliary cirrhosis (21). Accumulating evidence suggests that mitochondrial dysfunction plays a pivotal role in the pathogenesis of PBC. One of the hallmarks of PBC is the presence of AMAs that specifically target mitochondrial components, particularly PDC-E2, thereby contributing to bile duct damage (22) Additionally, oxidative stress, apoptosis, and metabolic dysregulation involving mitochondria significantly contribute to the disease process. Given these mitochondrial abnormalities in PBC, we performed a bioinformatics-based transcriptomic analysis to identify key mitochondrial-related genes associated with PBC.

The identification of DEGs, key modular genes, and mitochondria-related genes revealed that SHANK2 and TGM2 are key mitochondrial-related genes in PBC. Functional enrichment analysis revealed that they were involved in apoptotic clearance, oxidative phosphorylation, and nucleotide (pyrimidine and purine) metabolism. Apoptotic clearance is especially in PBC, and ineffective clearance of apoptotic cholangiocytes leads to the persistence of mitochondrial antigens and chronic immune activation. Similarly, oxidative phosphorylation impairment was associated with mitochondrial dysfunction in various liver diseases, which further supporting our findings. Both SHANK2 and TGM2 showed significantly higher expression levels in PBC patients compared to controls. SHANK2, a scaffold protein primarily studied in neurological disorders, has recently been implicated in immune modulation and cellular signaling (23). It was enriched in pathways related to T-cell receptor signaling and Wnt signaling, both of which have been reported to be dysregulated in PBC (24). TGM2, a multifunctional enzyme involved in the oxidative stress response, has been associated with oxidative phosphorylation, suggesting its role in mitochondrial function in PBC (25). Interestingly, TGM2 has also been implicated in extracellular matrix remodeling and fibrosis, potentially contributing to disease progression.SHANK2 is involved in the T-cell receptor and Wnt signaling pathways, which are intricately linked to mitochondrial energy metabolism and cellular development and differentiation processes. These complex interactions are likely to disrupt the normal dynamic balance of mitochondria, thereby adversely affecting immune cell function (23, 24). TGM2, a multifunctional enzyme, participates in oxidative stress responses and oxidative phosphorylation processes. On one hand, it may protect mitochondria from oxidative damage while regulating energy metabolism (25). On the other hand, when TGM2 function is impaired, it can lead to mitochondrial dysfunction, affecting the energy supply of immune cells. Moreover, its involvement in extracellular matrix remodeling is closely associated with disease progression (26) Mitochondria not only provide energy support to immune cells but also participate in immune regulation through various pathways (27). The reactive oxygen species (ROS) generated by mitochondria can activate the NLRP3 inflammasome to promote the release of pro-inflammatory cytokines such as IL-1β. Additionally, mitochondrial DNA (mtDNA) released into the cytoplasm can be recognized by the cGAS-STING pathway, triggering type I interferon responses. The activation of immune cells, including T cells and macrophages, relies on mitochondrial metabolic reprogramming, such as the shift from oxidative phosphorylation to glycolysis (28). These studies suggest that mitochondrial dysfunction may contribute to the development of autoimmune diseases by disrupting immune homeostasis (29, 30). In PBC, mitochondrial dysfunction breaks immune system homeostasis, and SHANK2 and TGM2, through their interactions with mitochondria, play critical roles in bridging immunity and mitochondrial function, opening up new avenues for understanding the pathogenesis of PBC.

In this study, immune infiltration analysis revealed significant differences in immune cell populations between PBC patients and controls. Notably, CD8+ T cells and resting NK cells were reduced in PBC patients, while M0 macrophages and resting memory CD4+ T cells were increased (31–33). The reduction in CD8+ T cells suggests that these cytotoxic T cells, which play a crucial role in the immune response to damaged bile ducts, may be functionally impaired or deficient in PBC. This dysregulation of CD8+ T cells has been previously been linked to autoreactive T cells contributing to bile duct injury, a hallmark of PBC (34). Similarly, the decrease in resting NK cells supports the hypothesis of immune dysfunction in PBC. NK cells are essential for immune surveillance and cytotoxicity, playing a protective role by targeting and eliminating infected or transformed cells (32). The observed reduction in resting NK cells suggests that their immune surveillance capacity may be compromised in PBC, facilitating disease progression.

An interesting finding was the elevation of M0 macrophages in PBC patients, which are immature, pro-inflammatory macrophages often seen in the early stages of immune activation. M0 macrophages have been implicated in inflammation and fibrosis in various chronic liver diseases, including PBC (35) These cells may contribute to the inflammatory microenvironment that drives the progression of bile duct damage in PBC. Furthermore, M2 macrophages, which are associated with tissue repair and fibrosis, were also significantly elevated in PBC compared to controls (36, 37). It has been shown that M2 macrophages play a crucial role in resolving inflammation and facilitating tissue remodeling. However, in the context of chronic inflammation, their activation can lead to excessive fibrosis (38). This study found that in PBC, there is a notable shift toward M2 macrophage polarization, which may contribute to the fibrosis observed in advanced stages of the disease. This finding is consistent with previous evidence suggesting an imbalance between M1 and M2 macrophages in autoimmune and chronic inflammatory conditions, where M2 macrophages promote fibrotic responses by secreting profibrotic cytokines and extracellular matrix components.

Regulatory network analysis revealed that SHANK2 is modulated by hsa-miR-665, while TGM2 is regulated by hsa-miR-6720-5p. Additionally, COLCA1, a gene previously associated with PBC susceptibility, was found to interact with these regulatory elements (39). The transcription factor TP53, a critical regulator of apoptosis and immune responses, was predicted to regulate SHANK2 (40, 41). These findings suggest that mitochondrial-related genes are under complex regulatory control in PBC, potentially linking metabolic dysfunction with immune dysregulation. Drug prediction analysis has identified vitamin A and calcium as potential therapeutic agents for PBC (42–45). Vitamin A deficiency has been documented in PBC patients, and supplementation may improve disease outcome. Similarly, abnormalities in calcium metabolism in PBC have been associated with osteoporosis and bone pain, making calcium supplementation a relevant therapeutic approach. Further experimental validation is required to determine the efficacy of these agents in the management of PBC.

To further validate the bioinformatics findings at both the protein and mRNA levels, we conducted immunohistochemical and RT-qPCR assays. Immunohistochemical analysis of liver tissue samples revealed that SHANK2 and TGM2 were significantly overexpressed in PBC patients compared to controls. Notably, SHANK2 expression showed a progressive increase from stage I to stage III of PBC, whereas TGM2 expression was elevated across all four histological stages, suggesting its continuous involvement throughout disease progression. These findings support the hypothesis that SHANK2 and TGM2 may not only participate in the early immune activation but also contribute to the chronic fibrotic remodeling observed in advanced PBC. Moreover, peripheral blood samples demonstrated significantly higher mRNA expression of both SHANK2 and TGM2 in PBC patients relative to healthy controls, reinforcing the systemic nature of their dysregulation. The consistent upregulation observed at both tissue and peripheral levels underscores the potential utility of SHANK2 and TGM2 as diagnostic biomarkers or therapeutic targets. These results corroborate the computational predictions and suggest that the dysregulated expression of SHANK2 and TGM2 may bridge mitochondrial dysfunction with immune dysregulation in PBC.

This study identified SHANK2 and TGM2 as key genes involved in the pathogenesis of PBC through transcriptome data and bioinformatics analysis. SHANK2 plays a role in immune regulation, while TGM2 is involved in mitochondrial function, including oxidative phosphorylation and metabolism. Both genes represent potential therapeutic targets for mitochondrial dysfunction in PBC. While these findings are promising, they require further validation. The role of SHANK2 and TGM2 in PBC should be confirmed using *in vitro* or animal models, and clinical validation in larger patient cohorts is necessary to assess their potential as biomarkers for diagnosis or prognosis. Future studies should focus on elucidating how SHANK2 and TGM2 interact with immune cells in PBC, particularly macrophages and T cells, and explore the regulatory networks that control their expression. This study performed transcriptomic analysis based on blood samples, with blood serving only as an “indirect surrogate” for liver pathology. Although the pathogenesis of PBC is associated with the abnormal activation of intrahepatic immune cells, the migration of these cells through the bloodstream allows the blood immune cell transcriptomic features to partially reflect the intrahepatic immune microenvironment. However, peripheral blood contains almost no cholangiocytes, which are the main target cells in PBC and whose damage and proliferation are core features of the disease. Therefore, the expression of cholangiocyte-related genes in the blood does not directly reflect intrahepatic pathological changes. Additionally, the gene expression patterns between the liver and blood microenvironments are significantly different. In the future, we plan to supplement our study with transcriptomic sequencing of liver biopsy samples and single-cell transcriptomic sequencing to further validate the reliability of our findings at both the gene and cellular levels and to precisely analyze the role of mitochondria-related genes in the intrahepatic pathological processes. Despite the limitations, our findings provide important insights into the molecular mechanisms of PBC and suggest potential avenues for targeted therapies.

## Data Availability

The datasets presented in this study can be found in online repositories. The names of the repository/repositories and accession number(s) can be found in the article/**Supplementary Material**.

## Supplementary material

Supplementary Table 1 Clinical and demographic information of 15 primary biliary cholangitis (PBC) patients and 15 healthy controls included in the training cohort for transcriptome sequencing.

Supplementary Table 2 Summary of sequencing quality metrics for 30 samples in the training cohort, including alignment rates (>90% for all samples) and base error rates (<1/1000).

Supplementary Table 3 Candidate hub genes identified by overlapping 1,905 differentially expressed genes (DEGs), 257 key module genes (from MEroyalblue), and 1,949 mitochondria-related genes (MRGs), yielding seven candidates: AURKA, C1QC, MAOA, RAD51, SHANK2, SPARCL1, and TGM2.
